# Binding deficits observed during short‐term memory tasks and advanced functions of everyday life might be signalling risk of abnormal ageing variants

**DOI:** 10.1002/alz70857_105081

**Published:** 2025-12-25

**Authors:** Joe Butler, Tamlyn J Watermeyer, Samuel O. Danso, Mario A A Parra, Binding in Neuropsychiatric Disorders (B.I.N.D) Group

**Affiliations:** ^1^ School of Psychology, University of Sunderland, Sunderland, United Kingdom; ^2^ National Institute of Health Applied Research Collaboration North East & Cumbria, Newcastle‐Upon‐Tyne, England, United Kingdom; ^3^ National Institute of Health Applied Research Collaboration North East & Cumbria, Newcastle‐upon‐Tyne, England, United Kingdom; ^4^ Edinburgh Dementia Prevention, Edinburgh, United Kingdom; ^5^ University of Northumbria, Newcastle upon tyne, England, United Kingdom; ^6^ School of Computer Science and Engineering, University of Sunderland, Sunderland, England, United Kingdom; ^7^ University of Edinburgh, Edinburgh, United Kingdom; ^8^ University of Strathcylde, Glasgow, Glasgow, United Kingdom

## Abstract

**Background:**

Poor performance on conjunctional or relational memory binding tasks is proposed as a neurocognitive marker for Alzheimer's Disease. However, it remains unclear i) how relational and conjunctional memory binding is affected by healthy ageing and ii) whether memory binding performance relates to advanced functions of everyday life underpinned by such memory constructs.

**Method:**

Younger (*n* = 41, 18–39 years, mean‐age = 23.56) and older (*n* = 41, 62–82 years, mean‐age = 73.78) adults completed reconstruction tasks measuring non‐spatial memory binding for integrated (conjunctive) or paired (relational) features. During encoding, relational trials involved three shape‐colour pairs, while conjunctive trials involved three coloured shapes. Immediately after, participants reconstructed feature pairs or integrated objects by choosing from a set of four coloured bubbles and a set of four line drawings of shapes and their corresponding colours. Scores reflected recognition of individual features and recall of feature pairings. Based on recent recommendations (Parra et al., 2024), participants were classified as weak or strong binders by contrasting scores drawn from the binding conditions relative to the single feature conditions using a 0.50 (∼25th percentile) cut‐off for the former. Participants also completed the Details of Function of Everyday Life (DoFEL) questionnaire.

**Result:**

ANCOVA revealed younger adults performed more accurately than older adults across all conditions (p < .001, η^2^ = .074). Individual features were more accurately recalled than paired/integrated features (p < .001, η^2^ = .398). The only significant interaction was that of age‐by‐binding type (*p* < .001, η^2^ = .031), which showed older adults were less accurate for paired/integrated features compared to younger adults (p < .001), while no age difference was observed for individual feature accuracy (p = .469), suggesting an age‐related binding decline. Relative to strong binders (*n* = 66), weak binders (*n* = 16) reported more functional impairments on the DoFEL (Mann‐Whitney test: p = .002, r = ‐0.46).

**Conclusion:**

Our results indicate that when ageing is associated with a selective decrease in memory binding abilities, further assessments should be carried out as such subtle memory impairments may underpin impairments in advanced functions of everyday life, thus pointing to an increased risk of being embarked on pathological ageing trajectories.

